# 肿瘤相关中性粒细胞在肺癌发生发展中的研究进展

**DOI:** 10.3779/j.issn.1009-3419.2025.101.02

**Published:** 2025-01-20

**Authors:** Xiaoyan LI, Jianjun ZHOU, Chaoting ZHAO, Yudi ZHANG

**Affiliations:** ^1^401420 重庆，重庆市綦江区中医院; ^1^Chongqing Qijiang Hospital of Traditional Chinese Medicine, Chongqing 401420, China; ^2^402760 重庆，重庆中医药学院; ^2^College of Combination of Chinese and Western Medicine, Chongqing University of Chinese Medicine, Chongqing 402760, China; ^3^400084 重庆，重庆医科大学附属康复医院; ^3^The Affiliated Rehabilitation Hospital of Chongqing Medical University, Chongqing 400084, China; ^4^402760 重庆，重庆中医药学院附属璧山医院; ^4^Bishan Hospital, Chonqqing University of Chinese Medicine, Chongqing 402760, China

**Keywords:** 肿瘤相关中性粒细胞, 肺肿瘤, 肿瘤微环境, 免疫, Tumor-associated neutrophils, Lung neoplasms, Tumor microenvironment, Immunity

## Abstract

肺癌在全球范围内是死亡率最高的恶性肿瘤类型。肿瘤微环境（tumor microenvironment, TME）是导致肺癌进展的关键因素，由肿瘤细胞、信号分子、成纤维细胞及免疫细胞等组成。其中，肿瘤相关中性粒细胞（tumor associated neutrophil, TAN）作为构成TME中免疫细胞的重要组成部分，其功能异常在肿瘤细胞的增殖、侵袭、血管生成和转移等环节发挥着多重作用，并与不良预后紧密相关。然而，TAN在肺癌中的作用机制研究较少。本综述通过阐述TAN的不同分型及其在肺癌发生、发展中的作用机制，旨在为研究肺癌的治疗靶点、开发新的药物提供更多的科学依据。

## Abstract (en)

Lung cancer is the malignant tumor with the highest mortality rate worldwide. The tumor microenvironment (TME) is a key factor in the progression of lung cancer, composed of tumor cells, signaling molecules, fibroblasts, immune cells, *etc*. Among them, tumor associated neutrophil (TAN), as an important component of immune cells in the TME, plays multiple roles in tumor cell proliferation, invasion, angiogenesis, and metastasis due to its aberrant function, and is closely associated with poor prognosis. However, there are limited researches on the mechanism of TAN in lung cancer. This review aims to provide more scientific basis for studying the therapeutic targets of lung cancer and developing new drugs by elucidating the different subtypes of TAN and their mechanisms of action in the occurrence and development of lung cancer.

肺癌是一种肺部最为常见的恶性肿瘤类型，其主要起源于气管、腺体或支气管黏膜。按组织病理学特征可分为两大类：非小细胞肺癌（non-small cell lung cancer, NSCLC）和小细胞肺癌（small cell lung cancer, SCLC）。其中NSCLC占所有肺癌病例的80%-85%，包括鳞状细胞癌、腺癌、大细胞癌以及其他较为罕见的病理亚型^[1]^。世界卫生组织国际癌症研究机构的数据^[2]^表明，肺癌是最常被诊断出的癌症，占全球癌症的1/8，其在癌症相关死因中占据主要位置，占全球癌症死亡人数的18.7%。在我国所有恶性肿瘤中，肺癌的疾病负担位居首位，新发病例和死亡人数分别占所有恶性肿瘤的22.0%和28.5%^[3]^。尽管目前采取了如靶向治疗、免疫疗法等各种创新治疗策略来治疗肺癌，但肺癌患者的5年生存率尚未达到理想水平，且治疗后的复发率和转移率依然居高不下^[4]^。近年来，越来越多的学者聚焦在调控肿瘤微环境（tumor microenvironment, TME）领域，以期防治肺癌的发生、发展及转移。

TME由肿瘤细胞、信号分子、免疫细胞、成纤维细胞及相关的细胞因子等组成，为肿瘤的发生和发展提供了必要的条件^[5]^。其中，肿瘤相关中性粒细胞（tumor associated neutrophil, TAN）作为构成TME中免疫细胞的重要组成部分，其功能异常在肿瘤细胞的增殖、侵袭、血管生成和转移等环节发挥着多重作用，并与不良预后紧密相关^[6]^。然而，TAN在肺癌中的作用机制研究较少。本综述旨在阐述TAN在肺癌发生发展中的作用及机制，为肺癌治疗策略提供新思路。

## 1 TAN概述

中性粒细胞被认为是抵抗感染的首要防线，在人体和小鼠中，分别占血液系统中白细胞总数的50%‐70%及10%‐25%^[7]^。鉴于中性粒细胞的生命周期相对较短，其在循环系统中的稳定存在依赖于机体分泌的粒细胞集落刺激因子（granulocyte colony stimulating factor, G-CSF）对骨髓前体细胞的持续性刺激作用^[8]^。在血液循环中，中性粒细胞大多时候处于静息状态，一旦迁移到组织中就会被激活^[9]^。中性粒细胞由骨髓产生而来，又被命名为多形核白细胞（polymorphonuclear cells, PMN），当炎症开始发生时，PMN对其反应十分迅速，大量PMN被细胞因子聚集至TME中转化为TAN。TAN是指肿瘤组织内呈浸润状态的中性粒细胞。肿瘤细胞释放特定的趋化因子，进而吸引血液循环中的中性粒细胞，促使其穿越血管壁，进而在肿瘤组织内部形成TAN^[10]^。在TME的调控下，一旦TAN被激活，它们可通过吸引其他白细胞的机制来导致炎症环境的复杂化^[11]^。研究^[12]^表明，TAN在肿瘤发展的多个方面扮演着关键角色，包括但不限于恶性转化、细胞外基质的改变、血管新生、肿瘤进展、细胞迁移以及免疫抑制等，这些因素与肺癌的预后紧密相关。

## 2 TAN在TME中的分型

在TME中，TAN可分为不同亚型。根据其功能不同可分为N1、N2和N0型；根据其离心后密度的差异可分为高密度中性粒细胞（high density neutrophil, HDN）以及低密度中性粒细胞（low density neutrophil, LDN）；根据其形态与表型特点将多核型髓源性抑制细胞（polymorphonuclear neutrophils myeloid-derived suppressor cell, PMN-MDSC）型也纳入了TAN的分型。不同亚型的TAN功能不同，且部分亚型TAN在TME中具有一定的可塑性。

### 2.1 N1/N2/N0型TAN

TAN在TME中的功能具有双向性，根据其功能不同分为以下3种亚型：（1）N1型TAN，又称抗肿瘤型TAN。N1型TAN可通过抗原提呈聚集并激活T细胞从而发挥抗肿瘤作用；其标志物包括CD15^high^、CD54^+^、CD86^+^、CD101^+^、CD117^+^、CD170^low^、人白细胞抗原-DR^+^（human leukocyte antigen-DR^+^, HLA-DR^+^）等。（2）N2型TAN，又称促肿瘤型TAN。N2型TAN可通过改变细胞外基质、促进肿瘤血管新生、肿瘤细胞迁移以及肿瘤细胞的免疫逃逸等多种机制，进而发挥促肿瘤作用；其标志物为血管内皮生长因子A（vascular endothelial growth factor A, VEGF-A）、碱性磷酸酶基因（alkaline phosphatase gene, ALPL）、基质金属蛋白酶9（matrix metalloproteinase 9, MMP-9）、CD11b^+^、趋化因子受体4（chemokine receptor 4, CXCR-4）、程序性死亡配体1（programmed death ligand 1, PD-L1）^+^和CD170^high^等。（3）N0型TAN，又称NM型TAN。N0型TAN被认为是一种介于N1型TAN和N2型TAN之间的状态^[13]^，其标志物为CD10^-^、CD11b^+^、CD66b^+^、CD84^+^、CD117^+^、内皮上的氧化低密度脂蛋白受体（receptor of oxiadized low desity lipoprotein, LOXI）和连接黏附样分子蛋白（junctional adhesion molecule-like protein, JAML）等。另外，单细胞测序结果^[14]^显示，TAN在TME中可进一步细分为5‐6个亚型，其中，在人类和小鼠模型中，*MMP-9*、S100钙结合蛋白A8（S100 calcium-binding protein A8, *S100A8*）（与肿瘤血管生成相关）和*S100A9*（可调控肿瘤细胞增殖）基因的表达产物具有高度一致性^[15]^。N2型TAN的基因表达产物可以超越物种间的差异，具有相对的稳定性，这为深入研究肺恶性肿瘤的发生和发展机制提供了关键线索。

TAN在TME中的功能具有一定的可塑性。在不同的TME中，TAN的功能可以被“交替激活”。研究^[16]^显示，肿瘤细胞可表达转化生长因子-β（transforming growth factor-β, TGF-β），促使TAN转化为N2型TAN，进而发挥促肿瘤作用。在肺癌小鼠模型中使用TGF-β抑制剂SMI6，抑制TGF-β信号传导可促使TAN转化为N1型TAN，使N1型TAN的数量增加，从而发挥抗肿瘤作用^[17]^。此外，I型干扰素（type I interferon, IFN-I）也可以诱导TAN向N1型TAN转化^[18]^。因此，TGF-β和IFN-I通过影响N1型和N2型TAN的转换，从而调节肺癌的发生发展，同时，这也揭示了TAN在TME中具有可塑性。在肿瘤发展的早期阶段，TAN以N1型为主，发挥抗肿瘤作用。然而，在肿瘤发展的晚期阶段，TAN以N2型为主，发挥促进肿瘤发展、侵袭和转移的作用^[19]^。以上研究为肿瘤防治提供了一个新的思路，即通过寻找激活N1型TAN和/或促进N2型转化为N1型TAN的策略来抑制肺癌进展。

### 2.2 LDN/HDN型TAN

根据其离心后密度差异，可将TAN分为具有抗肿瘤作用的HDN以及具有促肿瘤作用的LDN^[20]^，但目前尚缺乏用于区分它们的特异性表面标志物。Sagiv等^[21]^通过4T1小鼠乳腺肿瘤研究表明，在荷瘤小鼠体内，LDN型TAN表现出优先增殖的现象。通过形态学观察与功能验证，发现LDN型TAN由不成熟的LDN和成熟的LDN组成，后者同样具有免疫抑制特性。与N1型TAN和N2型TAN类似，在肿瘤早期，HDN型TAN占主导地位；随着肿瘤的发展，原本具有抗肿瘤作用的HDN型TAN会逐渐转化为促肿瘤生长的LDN型TAN，从而促进肿瘤发生发展^[22]^；进一步研究^[23]^显示，TGF-β亦可以介导HDN型TAN向LDN型TAN转化。以上结果显示，可通过调控TGF-β来减少LDN型TAN，从而发挥抗肿瘤作用。

### 2.3 MDSC型TAN

MDSC是一群来源于未成熟单核细胞和中性粒细胞的具有异质性的细胞群体。根据来源及形态特征，MDSC可分为单核细胞型MDSC（monocytic MDSC, M-MDSC）和PMN-MDSC，这两大亚群均具有强大的免疫抑制活性^[24]^。M-MDSC在形态与表型上与单核细胞相似，目前主要用CD11b^+^CD14^+^HLA^-^DR^-/low^CD15^-^来标记人类M-MDSC，用CD11b^+^Ly6G^-^Ly6C^high^来标记小鼠M-MDSC^[25]^。M-MDSC虽只占MDSC总数的10%-20%，但M-MDSC比PMN-MDSC具有更强的免疫抑制作用^[26]^。M-MDSC介导的免疫抑制机制包括产生抑制性细胞因子白细胞介素-10（interleukin-10, IL-10）和TGF-β^[27]^。在NSCLC的临床研究^[28]^中发现，M-MDSC可表达血管生成素受体酪氨酸激酶-2（angiopoietin-receptor tyrosine kinase-2, TIE2），进而抑制抗原特异性T细胞反应，这与患者的不良预后有关。

由于PMN-MDSC和TAN共同起源于骨髓，可分泌相同的生长因子和细胞因子，如G-CSF、IL-6和IL-17，因此很难明确区分PMN-MDSC和TAN，故将PMN-MDSC纳入TAN的一种亚型^[29]^。PMN-MDSC是MDSC最丰富的亚群^[30]^，其在人类标志物为CD11b^+^CD14^-^CD15^+^/CD116^+^CD14^+^CD66b^+^，在小鼠标志物主要有CD11b^+^Ly6G^+^Ly6C^low^^[25]^。PMN-MDSC可促进肿瘤的早期浸润，并在肺部和其他部位建立转移前生态位^[31]^，PMN-MDSC主要作用于T细胞，主要机制是产生活性氧（reactive oxygen species, ROS），使一氧化氮（nitric oxide, NO）和超氧化物反应产生过氧化亚硝酸盐（peroxynitrite, PNT）来消除CD8^+ ^T细胞，以抑制其与主要组织相容性复合体（major histocompatibility complex, MHC）的相互作用；PMN-MDSC还可以通过分泌细胞因子TGF-β来激活IL-4Ra-STAT6通路，从而发挥免疫抑制作用^[32]^。因此减少PMN-MDSC的产生、促进其失活或将成为改善肿瘤预后的有效手段。

## 3 TAN在肺癌发生发展中的作用机制

### 3.1 TAN在肺癌发生发展中的双向调节机制

#### 3.1.1 TAN依赖T细胞的双向调节作用

TAN可通过活化T细胞，进而实现对肿瘤细胞的杀伤效果。在早期肺癌患者体内，Singhal等^[33]^发现了一类独特的中性粒细胞亚群，它们具备抗原呈递细胞的特性，这些细胞通过释放CD54、CD86、肿瘤坏死因子超家族成员4（tumor necrosis factor superfamily member 4, TNFSF4）、TNFSF9等分子，发出共刺激信号，从而增强肿瘤特异性以及抗原非特异性T细胞反应。Eruslanov等^[34]^发现，在早期肺癌中，TAN能够分泌IL-6、IL-8、巨噬细胞炎症蛋白1（macrophage inflammatory protein 1, MIP-1）和MIP-1α等细胞因子，这些细胞因子能够激活T细胞，进而发挥抗肿瘤效应。此外，TAN还可以释放趋化因子，例如C-C基序趋化因子配体2（C-C motif chemokine ligand 2, CCL-2）、CCL3和CXCL-10，这些趋化因子有助于吸引T细胞向肿瘤组织集中^[35,36]^。研究^[33]^指出，在肺恶性肿瘤早期，特定表型的TAN，即CD11b^+^CD15^hi^CD10^-^CD16^int^/^low^型，可以诱导CD8^+^及CD4^+ ^T细胞向TME聚集并浸润，从而参与并促进抗肿瘤免疫反应。粒细胞-巨噬细胞集落刺激因子（granulocyte-macrophage colony-stimulating factor, GM-CSF）和干扰素-γ（interferon-γ, IFN-γ）能够刺激外周血中的中性粒细胞上调CD86以及HLA-DR的表达水平，展现出抗原提呈功能，增强T细胞的抗肿瘤免疫反应^[37]^。在对免疫治疗产生良好反应的肺癌小鼠模型中，发现了一类中性粒细胞亚群，其与IFN-γ激活基因相关^[38]^，且在免疫治疗中起到关键调节作用，增强了机体免疫细胞对肺癌细胞的识别和杀伤，提高了治疗效果。然而，TAN引发的T细胞活化仅在肿瘤早期阶段得以体现，随着肿瘤的进一步发展，此活化效应亦会逐渐减弱^[39]^。

TAN也可以通过抑制T细胞促进肿瘤转移。在肺癌组织里，TAN可以依靠释放趋化因子CCL4，使得巨噬细胞向肿瘤区域聚拢从而形成免疫抑制微环境，限制T细胞免疫反应，以此促进肿瘤转移^[40]^。此外，TAN亦能通过与其他免疫细胞发生相互作用来影响肿瘤免疫逃逸的机制^[41]^。在肺癌早期阶段，TAN可促进T细胞的免疫反应，抑制肿瘤进展，发挥抗癌作用；然而随着疾病进展，TAN逐渐转为抑制T细胞的免疫反应，促进肿瘤进展，发挥促癌作用。因此，TNA对肺癌的双重调节作用是通过调节T细胞的活性实现的。

#### 3.1.2 TAN依赖中性粒细胞胞外诱捕网（neutrophil extracellular traps, NETs）的双向调节作用

TAN可以通过形成NETs对肿瘤发挥双向调节作用，不仅能抑制肿瘤生长，也能促进肿瘤发展。NETs是中性粒细胞在激活后释放到胞外的网状结构，由双链DNA、组蛋白、中性粒细胞弹性蛋白酶、髓过氧化物酶、MMP-9等颗粒蛋白组成^[42]^。NETs能召集TME中的树突状细胞从而抑制血管生成，还能激活T细胞，并与Toll样受体2（Toll-like receptor 2, TLR-2）相互作用，诱导转录激活蛋白3磷酸化，促进Th17分化，发挥抗肿瘤作用^[43,44]^。目前对TAN依赖NETs的抗肺癌作用机制研究相对较少，一些研究者提出假说，认为NETs可能是通过激活免疫反应或直接杀伤肺癌细胞从而发挥抗肿瘤作用。

TAN可促进NETs的形成，而NETs的存在亦会进一步促进肿瘤进展，研究^[45]^指出，NETs水平的升高与肿瘤的进展存在正向相关性。NETs可直接促进肿瘤细胞的渗出、增殖、侵袭和迁移，并通过激活休眠癌细胞、促进癌细胞的恶性生物过程、诱导炎症因子生成、参与转移前生态位的形成等方式，促进肿瘤细胞的生长和转移^[46]^。研究^[47]^显示，长链非编码核糖核酸（long non-coding RNA, lncRNA）MIR503HG的表达在经NETs刺激的肺癌细胞中高度失调，NETs可通过下调lncRNA MIR503HG表达来激活核因子κB（nuclear factor kappa B, NF-κB）/核苷酸结合寡聚化结构域样受体热蛋白结构域相关蛋白3（NOD-like receptor thermal protein domain associated protein 3, NLRP3）信号传导途径，NLRP3炎性小体的过度活化会增强肿瘤侵袭和转移能力。肺癌患者体内NETs的标志物与补体C5a水平显著相关，C5a促进了PMN-MDSC中HMGB1受体TLR4和晚期糖基化终末产物受体（receptor for advanced glycation end products, RAGE）的表达，诱导NETs形成，并显著增强肺癌细胞的侵袭能力^[48]^。

TAN的促癌作用与炎症也密不可分，TAN是癌症和炎症关系中的重要媒介。一项包含近50万名成年参与者的前瞻性队列研究^[49]^指出，肺癌的发病风险与白细胞计数呈现正向关联，且此关联主要体现在中性粒细胞数量的上升。此外，在肺癌小鼠模型中，已证实NETs存在于微血管中，并能捕获肿瘤细胞DNA，从而促进肿瘤转移^[48]^。NETs将肿瘤细胞和靶器官内皮细胞连接在一起，发挥促肿瘤作用。NETs在肿瘤转移的作用机制与其在全身感染中的病理机制有着密切联系，NETs可以促进炎症反应，进一步加剧TME的恶化，从而为肿瘤细胞的转移提供有利条件。鉴于NETs在肿瘤转移中的关键作用，它们可以成为肿瘤治疗的靶向目标。

因此，NETs可刺激免疫系统反应抑制肿瘤细胞生长或直接杀伤肿瘤细胞，从而发挥抗肿瘤作用，亦可通过直接促进肿瘤细胞的渗出、增殖、侵袭和迁移等方式，促进肿瘤细胞发生免疫逃逸和转移而发挥促肿瘤作用。肺癌小鼠模型及肺癌患者中，TAN可以通过促进NETs形成发挥双重作用，NETs或可成为靶向治疗肺癌的新策略。

### 3.2 TAN的直接抗肿瘤机制

TAN直接杀伤肿瘤细胞的主要方式是分泌ROS，而ROS主要是依赖于TRPM2离子通道发挥抗肿瘤效应，因此，该机制仅对高表达TRPM2的癌细胞展现出显著的杀伤作用^[50]^。另外，TAN也能释放肿瘤坏死因子（tumor necrosis factor, TNF）及NO，进一步参与肿瘤的抑制过程^[51]^。Cui等^[52]^通过检测PMN活力时发现，PMN对人体正常细胞影响不大，但对肺癌、脑胶质瘤等多种恶性肿瘤均有一定的杀伤作用。研究^[53,54]^发现，在荷瘤小鼠脾脏中，随着疾病进展PMN获得抗原提呈能力，并逐渐被PMN-MDSC取代，从而发挥抗肿瘤作用。除此之外，TAN亦可借助抗体依赖的细胞介导的细胞毒作用（antibody dependent cellular cytotoxicity, ADCC）来实现对肿瘤细胞的杀伤作用。中性粒细胞通过“胞啃”的方式杀伤与抗体结合的肿瘤细胞，其主要通过干扰SIRP-α与CD47相互作用的吞噬抑制信号通路来增强“胞啃”作用，发挥ADCC效应^[55]^。综上。TAN可以通过分泌ROS、释放NO和TNF及依赖ADCC效应直接杀伤肿瘤，发挥抗肿瘤作用。

### 3.3 TAN的直接促肿瘤机制

在肿瘤转移过程中，血管生成属于极为关键的一个环节，血管基质蛋白的表达与肿瘤细胞的增殖和转移存在紧密的关联。存在于肿瘤血管基质分子中的肿瘤组织蛋白等成分可被N2型TAN自主分泌的MMP-9降解，致使血管基质分子结构被改变，正常组织屏障被破坏，从而推动新生血管上皮的形成，使得肿瘤细胞的转移能力和体外浸润性有所增强^[56]^。TAN不仅可通过调控VEGF和MMP-9的表达来促进肺癌新生血管的生成，还可通过抑制多聚腺苷二磷酸核糖聚合酶-4促进其新生血管的生成，增强肺癌的发展及转移能力^[57]^。此外，在肿瘤异种移植模型中，G-CSF可促进TAN募集，分泌前动力蛋白-2，调控VEGF，诱导肿瘤血管生成，提高肿瘤细胞的体外浸润能力及迁移能力，从而促进肿瘤的发展及转移^[58,59]^。G-CSF对TAN的功能具有调节作用，能够促进TAN形成NETs、产生NO及ROS，并促使表达PD-L1。因此G-CSF类药物作为一种关键的抗肿瘤辅助治疗手段，在临床应用中被广泛用来预防和处理由化疗药物引发的中性粒细胞减少症。

综上，本文重点阐述了TAN的不同分型及在肺癌发生、发展中的作用机制（表1）。TAN是肺癌TME中的主要免疫细胞类型，参与构建高度免疫抑制的环境，调控肿瘤生长和扩散，已成为影响肺癌发生与进展的关键因素，与肺癌预后密切相关^[60]^。另外，NETs已被证明可调节肺癌发病机制和进展，靶向调控NETs的形成亦可能成为一种有效的治疗策略。TAN在肿瘤学中作用的复杂性在于亚群的多样性及其在肿瘤环境影响下的可塑性，因此TAN分型和功能的可塑性或将成为肺癌干预的重要靶点，促使TAN向抗肿瘤表型转化，这将有助于开发新的治疗策略。然而，目前关于TAN在肿瘤尤其是肺癌中作用机制的相关研究仍然有限，缺乏多方面的实验证据。随着研究的深入开展，TAN在抗肿瘤治疗方面的价值将逐渐体现，是潜在的抗肿瘤治疗有效靶点。

**表1:** TAN的分型及其在肺癌中的作用机制

Classification standards	Classification	Effect	Mechanisms
Function	N0	Bidirectional regulation	The state between N1 type TAN and N2 type TAN, with bidirectional regulation.
	N1	Antitumor	1. Anti-tumor mechanism with T cells dependence: (1) release of CD54, CD86, TNFSF4, TNFSF9 and other molecules, send out costimulatory signals, and enhance tumor-specific and antigen-nonspecific T cell responses^[33]^; (2) Secreting cytokines such as IL-6, IL-8, MIP-1 and MIP-1α to activate T cells^[34]^; (3) GM-CSF and IFN-γ stimulate peripheral neutrophils to up-regulate the expression levels of CD86 and HLA-DR, and enhance the antigen presenting function^[37]^; (4) CD11b^+^CD15^hi^CD10^-^CD16^int/low^ TAN induced CD8^+^ and CD4^+^ T cells to aggregate towards TME^[33]^. 2. Anti-tumor mechanism with NETs dependence: NETs convene DC in TME to inhibit angiogenesis, activate T cells, interact with TLR-2, induce phosphorylation of transcription-activating protein 3, and promote Th17 differentiation^[43,44]^. 3. Direct anti-tumor mechanism of TAN: (1) secreting ROS, TNF and NO to exert anti-tumor effects^[50,51]^; (2) ADCC effect^[55]^.
	N2	Tumor promotion	1. The tumor-promoting mechanism with T cells dependence: release of chemokine CCL4, causing macrophages to accumulate in the tumor area, forming an immunosuppressive microenvironment, limiting T cell immune response, and promoting tumor metastasis^[40]^. 2. Direct tumor promoting mechanism of TAN: regulate the expression of VEGF and MMP-9, or inhibit polyadenosine diphosphoribose polymerase-4, so as to promote the formation of neovascularization and enhance the development and metastasis of lung cancer^[57]^.
Morphology and phenotype	PMN-MDSC	Tumor promotion	1. The tumor-promoting mechanism with T cells dependence: secretion of cytokines, such as TGF-β, plays an immunosuppressive function by activating the IL-4Ra-STAT6 pathway^[32]^. 2. Tumor-promoting mechanism with NETs dependence: (1) NETs activate the NF-κB/NLRP3 pathway by down-regulating the expression of lncRNA MIR503HG, and overactivation of NLRP3 inflammasome may promote tumor invasion and metastasis^[47]^; (2) NETs capture tumor cell DNA in microvessels, thus promoting tumor metastasis^[48]^.
Density	LDN	Tumor promotion	1. Mature LDN has immunosuppressive properties. 2. With the progression of tumor, anti-tumor HDN TAN will be transformed into pro-tumor LDN TAN, thus promoting tumor occurrence and development^[22]^.
	HDN	Antitumor	

TAN: tumor associated neutrophil; PMN-MDSC: polymorphonuclear neutrophils myeloid-derived suppressor cell; NETs: neutrophil extracellular traps; LDN: low density neutrophils; HDN: high density neutrophils; TNFSF4: tumor necrosis factor superfamily member 4; TNFSF9: tumor necrosis factor superfamily member 9; IL-6: interleukin-6;IL-8: interleukin-8; MIP-1: macrophage inflammatory protein 1; MIP-1α: macrophage inflammatory protein 1α; IFN-γ: interferon-γ; HLA-DR: human leukocyte antigen-DR; TME: tumor microenvironment; TLR-2: Toll-like receptor 2; ROS: reactive oxygen species; TNF: tumor necrosis factor; NO: nitric oxide; ADCC: antibody dependent cellular cytotoxicity; CCL4: C-C motif chemokine ligand 4; TGF-β: transforming growth factor-β; NF-κB: nuclear factor kappa B; NLRP3: NOD-like receptor thermal protein domain associated protein 3; lncRNA: long non-coding RNA; VEGF: vascular endothelial growth factor; MMP-9: matrix metalloproteinase 9; GM-CSF: granulocyte-macrophage colony-stimulating factor; DC: dendritic cell.
